# *Notes from the Field:* Increase in Diagnoses of Human Parvovirus B19–Associated Aplastic Crises in Children and Adolescents with Sickle Cell Disease — Atlanta, Georgia, December 14, 2023–September 30, 2024

**DOI:** 10.15585/mmwr.mm7347a5

**Published:** 2024-11-28

**Authors:** Marianne E.M. Yee, Grace G. Kalmus, Ashwin P. Patel, Jason N. Payne, Amy Tang, Beatrice E. Gee

**Affiliations:** ^1^Aflac Cancer and Blood Disorders Center, Children’s Healthcare of Atlanta, Atlanta, Georgia; ^2^Department of Pediatrics, Emory University School of Medicine, Atlanta, Georgia; ^3^Department of Pediatrics, Morehouse School of Medicine, Atlanta, Georgia.

**Summary**

**What is already known about this topic?**

Human parvovirus B19 (B19) can cause transient aplastic crises in persons with chronic anemia, including sickle cell disease (SCD).

**What is added by this report?**

In a large SCD center in the southeastern United States, the incidence rate of B19-associated aplastic crisis was 3.6 times higher in the first 9 months of 2024 compared with the overall rate during 2010–2023.

**What are the implications for public health practice?**

Health care providers should be aware of increased B19 activity in 2024 and consider B19 infection in persons with SCD presenting with anemia and reticulocytopenia because this population might require urgent blood transfusion to reduce the risk for severe complications.

Human parvovirus B19 (B19) is a common viral infection transmitted by respiratory droplets. Although B19 infection is typically mild in healthy persons, persons with sickle cell disease (SCD) can experience severe anemia from impairment of red blood cell production (*1*–*3*).

In December 2023, a child aged 10 years with SCD (child A) died unexpectedly at home, with no preceding fever or symptoms. Limited laboratory testing showed hematocrit <15% (normal = 37%–45%); testing for B19 was not performed. Massive splenomegaly was noted on autopsy, consistent with splenic sequestration crisis associated with SCD. Six days later, child A’s sibling (child B), aged 14 years, who also has SCD, was confirmed to have acute B19 infection. Child B’s laboratory evaluation showed hemoglobin <6 g/dL (normal = 12.0–16.0 g/dL) and reticulocyte count 7.0 × 10^9^/L (normal = 40–102 × 10^9^/L), consistent with aplastic crisis. Child B received a red blood cell transfusion and recovered without complications. Data from the Sickle Cell Clinical Database of Children’s Healthcare of Atlanta (CHOA) were reviewed to describe recent trends in B19 infection among children with SCD. This study was approved by the Institutional Review Board of CHOA.

## Investigations and Outcomes

At CHOA, patients with SCD are presumed to have aplastic crisis and are tested for B19 if laboratory evaluation shows a decrease in hemoglobin ≥1 g/dL from baseline with reticulocytopenia. Examination of data from CHOA’s Sickle Cell Clinical Database identified 55 cases of B19 infection with aplastic crisis in children with SCD during December 2023–September 30, 2024 (two in 2023 [child B and another, unrelated child] and 53 in 2024). During January–September 2024, the incidence of B19 infection associated with aplastic crisis among all pediatric patients with SCD who had a clinical encounter at CHOA during that calendar year was 35.6 per 1,000 patient-years, 3.6 times higher than that during 2010–2023 (7.78 per 1,000 patient-years) (Figure). Increased numbers of B19-associated aplastic crisis cases and incidence occurred in 2014 (34 cases; 19.2 per 1,000 patient-years) and 2019 (30 cases; 14.7 per 1,000 patient-years). Based on clinical suspicion, during 2010–2023, a median of 2.9% of all patients with SCD with health care encounters at CHOA were tested for B19 per year (range = 1.4%–5.7%); in 2024, 6.8% of patients were tested (Supplementary Figure, https://stacks.cdc.gov/view/cdc/170361).

**FIGURE:**
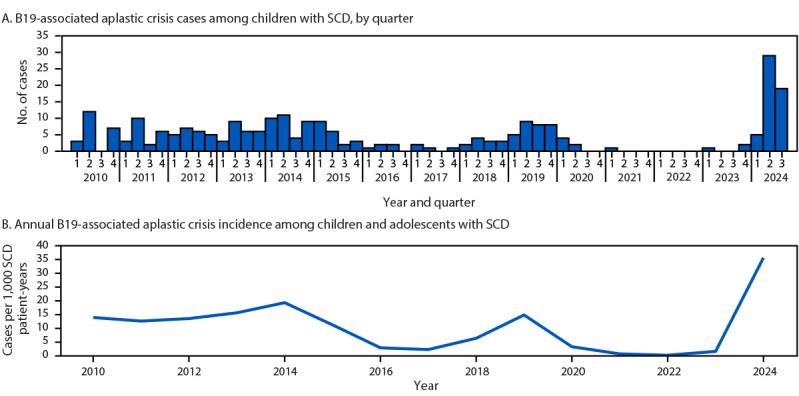
Human parvovirus B19–associated aplastic crisis cases (A) and incidence (B) identified among children and adolescents with sickle cell disease — Children’s Healthcare of Atlanta, Atlanta, Georgia, January 1, 2010–September 30, 2024 **Abbreviations:** B19 = human parvovirus B19; SCD = sickle cell disease.

The median patient age at infection since December 2023 was 10.1 years, compared with 7.7 years during 2010–January 2023 (p = 0.004).* Common signs and symptoms included pain (78%), fever (62%), fatigue (31%), and respiratory symptoms (26%). The median decline in hemoglobin from baseline to nadir was 3.6 g/dL (IQR = 3.0–4.8). Complications included acute chest syndrome^†^ (27%), splenic sequestration^§^ (11%), stroke (3.6%), and nephrotic syndrome (1.8%). Forty-three (78%) patients received red blood cell transfusions. Other than child A, in whom a diagnosis of B19 was not confirmed, no other patients died with B19 infection.

## Preliminary Conclusions and Actions

During 2024, an increased incidence of B19-associated aplastic crisis was observed among patients with SCD at one pediatric health care system in Atlanta, compared with incidence during 2010–2023. Among SCD patients with B19 infection, the most common initial signs of infection include anemia and reticulocytopenia. Health care providers should be aware of the risk for complications of B19 infection in children with SCD and have a low threshold for testing when there is clinical suspicion of aplastic crisis. Children and adolescents with SCD and B19 infection should be monitored for complications; early red blood cell transfusion might prevent serious adverse outcomes.

## Footnotes

* Wilcoxon rank-sum test.

† A vaso-occlusive complication in the pulmonary vasculature.

§ A sudden enlargement of the spleen caused by trapping of sickled red blood cells, resulting in reduction in blood volume and hemoglobin.
